# Fractionated **γ**-irradiation renders tumour cells more responsive to apoptotic signals through CD95

**DOI:** 10.1038/sj.bjc.6690585

**Published:** 1999-08

**Authors:** M A Sheard, P H Krammer, J Zaloudik

**Affiliations:** Masaryk Memorial Cancer Institute, Zluty kopec 7, 656 53 Brno, The Czech Republic; German Cancer Research Center, Heidelberg, D-69120, Germany

**Keywords:** CD95, apoptosis, ionizing radiation, carcinoma, p53

## Abstract

Signals through the CD95 surface receptor can specifically induce apoptosis. Some tumour cell lines are sensitive to CD95 signals, and insensitive cells can be converted to a sensitive phenotype if given appropriate treatment. To determine whether the apoptotic response of tumour cells to signalling through CD95 might be enhanced by ionizing irradiation, carcinoma cells were treated with either single-dose or fractionated γ-irradiation. The response to treatment with an agonist anti-CD95 antibody was enhanced by pretreatment with either a single large dose or daily fractionated radiation. Fractionated irradiation induced cumulative and prolonged up-regulation of CD95 expression in cell lines bearing functional p53. Since two of four cell lines exhibiting heightened responsiveness to CD95-mediated signals following fractionated irradiation express mutant p53 and displayed little or no up-regulation of CD95, enhanced responsiveness did not correlate with p53 status and CD95 up-regulation. Continuous inhibition of CD95/CD95–ligand interactions during fractionated irradiation provided no protective effect to cells, arguing that autologous CD95/CD95–ligand interactions did not contribute to the direct lethal effect of irradiation. We conclude that fractionated γ-irradiation provides an extended period of time when carcinoma cells are more responsive to CD95-mediated signals in vitro. © 1999 Cancer Research Campaign


*British Journal of Cancer* (1999) **80**(11), 1689–1696
© 1999 Cancer Research Campaign
Article no. bjoc.1999.0585

### Fractionated γ-irradiation renders tumour cells more responsive to apoptotic signals through CD95

**MA Sheard[1], PH Krammer[3] and J Zaloudik[1]**

[1]Masaryk Memorial Cancer Institute, Zluty kopec 7, 656 53 Brno, The Czech Republic; [2]German Cancer Research Center, D-69120 Heidelberg, Germany

**Summary** Signals through the CD95 surface receptor can specifically induce apoptosis. Some tumour cell lines are sensitive to CD95 signals, and insensitive cells can be converted to a sensitive phenotype if given appropriate treatment. To determine whether the apoptotic response of tumour cells to signalling through CD95 might be enhanced by ionizing irradiation, carcinoma cells were treated with either single-dose or fractionated γ-irradiation. The response to treatment with an agonist anti-CD95 antibody was enhanced by pretreatment with either a single large dose or daily fractionated radiation. Fractionated irradiation induced cumulative and prolonged up-regulation of CD95 expression in cell lines bearing functional p53. Since two of four cell lines exhibiting heightened responsiveness to CD95-mediated signals following fractionated irradiation express mutant p53 and displayed little or no up-regulation of CD95, enhanced responsiveness did not correlate with p53 status and CD95 up-regulation. Continuous inhibition of CD95/CD95–ligand interactions during fractionated irradiation provided no protective effect to cells, arguing that autologous CD95/CD95–ligand interactions did not contribute to the direct lethal effect of irradiation. We conclude that fractionated γ-irradiation provides an extended period of time when carcinoma cells are more responsive to CD95-mediated signals in vitro.

**Keywords:** CD95; apoptosis; ionizing radiation; carcinoma; p53

CD95 (Fas, APO-1) is a death receptor in the plasma membrane that specifically triggers apoptosis when appropriately stimulated on the surface of sensitive cells (Krammer et al, 1994; Nagata, 1997). The ligand for CD95 (CD95-L) is expressed by activated cytotoxic T lymphocytes (Suda et al, 1995) and natural killer cells (Eischen et al, 1996; Oshimi et al, 1996). Signals through CD95 can contribute to tumour cell death induced by these immunologic killer cells in vitro (Mori et al, 1997; Williams et al, 1997). Recent reports have shown that treatment with cytotoxic drugs can up-regulate tumour cell expression of CD95, and that these cells coincidentally become more responsive to CD95 signalling (Fulda et al, 1997; Müller et al, 1997; Williams et al, 1997).

Multiple mechanisms are employed by tumour cells to develop resistance to CD95 signalling. Reduction or loss of CD95 on the cell surface has been reported to desensitize leukaemia cells to treatment with an agonistic anti-CD95 antibody (Ab) (Landowski et al, 1997*a*; Martinez-Lorenzo et al, 1998). Mutation of the cytoplasmic region of the *cd95* gene has been detected in a subset of multiple myeloma patients (Landowski et al, 1997*b*). Disruption of the downstream protein caspase-8 (FLICE) in the CD95 signalling pathway by overexpression of the cellular FLICE-inhibitory protein (cFLIP) results in resistance to the cytotoxic effects of both CD95 and TNF-R1; in the first report of its kind, both melanoma cell lines and malignant melanoma tumours were reported to overexpress cFLIP, and were resistant to signals through CD95 (Irmler et al, 1997). However, cells that are resistant to CD95-mediated signalling can be sensitized by treatment with cycloheximide (CHX), actinomycin D, brefeldin A, or interferon (INF)-γ (von Reyher et al, 1998), although the mechanism for sensitization remains unclear.

Mammalian cells respond to ionizing irradiation in a variety of ways, depending on the type and condition of the cell. In response to DNA damage, the tumour suppressor protein p53 activates two opposing cellular pathways, one resulting in cell-cycle arrest and one triggering apoptosis (reviewed in Ashkenas and Werb, 1996). Thymocytes are highly sensitive to γ-irradiation and die rapidly by apoptosis in a p53-dependent fashion following a single dose of 1 Gy (Clarke et al, 1993; Lowe et al, 1993). Carcinoma cells are more resistant to the effects of ionizing irradiation; in one recent study, a single large dose of γ-rays (> 12 Gy) induced measurable cell death in some, but not all carcinoma cell lines (Zhan et al, 1994). Human carcinoma patients typically cannot be irradiated with high doses; instead, clinical irradiation is routinely fractionated into relatively small, daily doses (Lichter and Lawrence, 1995).

We have previously reported that treatment of tumour cells with ionizing radiation induces up-regulation of surface CD95 expression, but only in cells exhibiting wild-type p53 activity (Sheard et al, 1997). To evaluate whether ionizing irradiation might contribute to tumour cell vulnerability by increasing the response to CD95-mediated apoptotic signals, human epithelial tumour cells were treated with high-dose or fractionated low-dose γ-irradiation and examined for susceptibility to CD95-mediated apoptosis.

*Received 12 October 1998*
*Revised 12 January 1999*
*Accepted 4 March 1999*

*Correspondence to:* MA Sheard

### MATERIALS AND METHODS

### Cell cultures

Human colorectal carcinoma (HCT116 and HT29), breast adenocarcinoma (MCF-7, BT549, MDA-MB231), breast ductal carcinoma (T47D), cervical carcinoma (HeLa) and osteosarcoma (SAOS-2) cells were cultured in Dulbecco's modified Eagle's medium (DMEM). For experiments involving a single dose of radiation, cells were plated at sub-confluence in 75 $cm^2$ tissue culture flasks (Nalge Nunc International, Denmark) 1 day prior to irradiation, and maintained in 10% fetal calf serum (FCS). During dose-fractionated experiments, cell cultures consistently expanded to confluency, regardless of low starting concentrations; fresh medium containing 1% FCS (except where noted) was supplied every 3 days, 8 h after irradiation (to avoid conflict between transient post-irradiation p53 activity and serum-derived mitogenic signals), and 1 day before anti-CD95 treatment (to avoid conflict between CD95-induced apoptotic signals and mitogenic signals from freshly-administered serum). The status of p53 has been reported for all cell lines studied.

### Radiation treatment

Cell lines were γ-irradiated from a $^{60}$Co source (dose rate 1.30 Gy $min^{-1}$). To assess basal levels of CD95, equivalent cell cultures were mock-treated by removing from incubators and tightening caps during the irradiation periods. For fractionated irradiation, cells were exposed daily to 2 Gy for 8 consecutive weekdays, Tuesday to Friday in the first week, Monday to Thursday in the second week.

### Induction and inhibition of CD95-mediated apoptosis

Eight hours after completion of irradiation, agonist anti-CD95 IgM monoclonal Ab (clone CH11, Immunotech, Westbrook, ME, USA) was added directly to cell cultures for a final concentration of 1 μg $ml^{-1}$ (in CD95-resistant cells) or 0.2 μg $ml^{-1}$ (in partially-sensitive cells), and cells were incubated for 44 h (in single-dose experiments) or 20 h (in fractionated-dose experiments) in 5% carbon dioxide at 37°C prior to harvesting. Prior to addition of agonist Ab, CD95-resistant cells were pretreated with cycloheximide (10 μg $ml^{-1}$) (SIGMA, St Louis, MO, USA) for 30 min. To inhibit CD95-mediated apoptosis, the previously described antagonistic $F(ab')_2$ Ab fragments (anti-APO-1) (Dhein et al, 1995) were added at 1 μg $ml^{-1}$ 30 min before addition of agonist Ab in single-dose experiments, or immediately after every exchange of medium when examining potential CD95/CD95-L interactions during fractionated irradiation.

### DNA content analysis

For measurement of DNA content, adherent and floating cells were washed separately, pooled together, and incubated for at least 4 h at 4°C in 400 μl DNA staining solution (50 μg $ml^{-1}$ propidium iodide, 0.1% Triton X-100, 0.07% RNase, 0.1% sodium chloride). Cells were analysed by flow cytometry in their staining solution diluted to 0.8 ml with modified Hank's balanced salt solution (HBSS). A 630 nm band-pass filter was used to collect red fluorescence. In each histogram, a gate was applied to the lowest spectrum of the linear FL3 channel at the distinct border between apoptotic cells and subcellular debris. Data were acquired in linear mode instead of logarithmic mode to reduce the negative aspect associated with setting subjective gates on forward and side-scatter parameters when delineating between late apoptotic events and subcellular debris (late apoptotic cells are a source of debris-related noise), which was more problematic when very low-staining FL3 signals were included into analyses using a logarithmic scale.

### TdT-mediated dUTP nick end labelling (TUNEL)

TUNEL technique was performed using a kit (Boehringer Mannheim, Mannheim, Germany) according to instructions provided by the manufacturer, with several modifications. Briefly, harvested cells (adherent plus floating) were transferred to 'eppendorf' tubes and washed twice in modified HBSS containing 0.2% bovine serum albumin (BSA) and 0.1% sodium azide ($NaN_3$); to avoid excessive cell loss during the many washing steps, all washes were performed by centrifuging cells for 8 min at 300 ***g*** (in early washes), aspirating supernatants to a minimal volume using individual disposable pipettes, and adding a few drops of medium to each tube for adequate volume during subsequent vortexing. Cells were fixed in 4% paraformaldehyde for 30 min at room temperature. After two washes in phosphate-buffered saline (PBS), cells were resuspended in permeabilization solution (0.1% Triton X-100, 0.1% sodium citrate) for 5 min and washed twice (spin rate was henceforth increased to 400 G to improve cell yield). To relieve compaction of DNA by histones, cells were incubated in proteinase K (20 μg $ml^{-1}$) at 37°C for 20 min. Following two washes, cells were incubated in the provided staining solution for 2 h at 37°C, washed twice, and analysed by flow cytometry.

### Determination of surface protein expression

Cells were harvested with 0.1% trypsin, 0.25% EGTA in modified HBSS without calcium or magnesium, and maintained in modified HBSS containing 0.2% BSA and 0.1% $NaN_3$. Cells were washed twice, stained with fluorescein isothiocyanate (FITC)-conjugated monoclonal anti-human CD95 (clone UB2) or FITC-IgG1 isotype control Ab (Immunotech, Westbrook, ME, USA), washed twice, and analysed by flow cytometry using an EPICS XL (Coulter, Hialeah, FL, USA). The voltage applied to the FL1 channel was selected for each treatment-group in a given cell type so that the mean fluorescence intensity (MFI) of irrelevant Ab-treated controls was similar. Indices of MFI values were calculated using the formula: MFI Index = (MFI of anti-CD95 Ab-stained cells/MFI of irrelevant Ab-stained cells). Dead cells and debris were excluded according to their increased staining with propidium iodide and low forward scatter properties. At least 10 000 viable cells were interrogated for each sample.

### RESULTS

### Response to signals through CD95 after a single large dose of γ-radiation

To evaluate whether ionizing radiation can sensitize tumour cells to apoptotic signalling through CD95, HCT116 colorectal carcinoma cells were treated with 16-Gy γ-rays. Eight hours later, irradiated and control cells were treated with CHX, with an agonistic cross-linking anti-CD95 Ab, or both, and then incubated

© 1999 Cancer Research Campaign

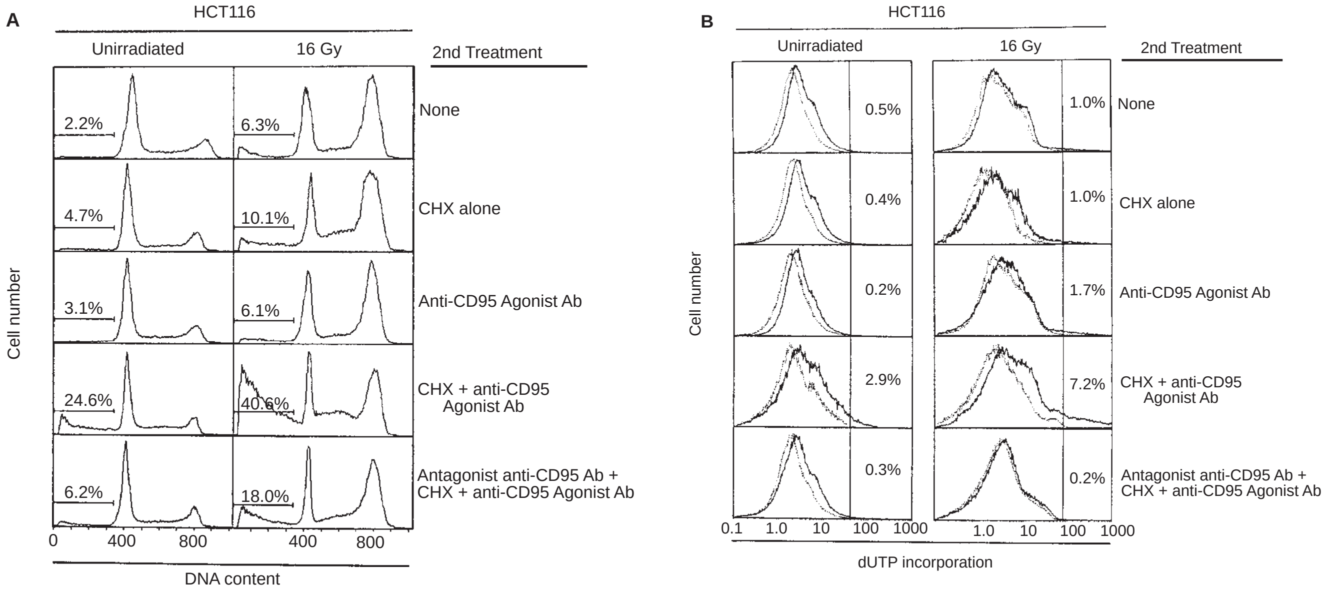

**Figure 1** Effect of signals through CD95 on viability of tumour cells γ-irradiated with a single large dose. Eight hours after γ-irradiation, HCT116 cells were pretreated with CHX alone (10 μg $ml^{-1}$) for 30 min, treated with agonist anti-CD95 Ab alone (1 μg $ml^{-1}$), or treated with both CHX and then agonist Ab. To evaluate the specificity of killing, controls were incubated with an antagonist anti-CD95 Ab 30 min before addition of agonist Ab. After 44 h, cells were harvested and stained for analyses by flow cytometry, as described in Materials and Methods. (**A**) DNA content was analysed for individual cells by staining DNA with propidium iodide. Peaks representing cells in G1 phase of the cell-cycle are located at channel 400. The percentage of events detected with a DNA-content less than in G1 phase of the cell-cycle is given in each panel. Similar results were obtained in four independent experiments. (**B**) DNA strand breaks were detected by TUNEL staining. The percentage of specific staining with dUTP-FITC is given in each panel and was calculated as follows: (% positive in TUNEL reaction) − (% positive in controls stained with TdT only)

another 2 days. DNA content was evaluated for each cell by staining with propidium iodide and analysing by flow cytometry. Only a low percentage of untreated HCT116 cells contained a DNA content less than observed in G1 phase of the cell-cycle (sub-G1); (we avoid the common term 'subdiploid', since many tumour cell lines in G1 phase contain an aneuploid number of chromosomes). Treatment of unirradiated control cells with agonist anti-CD95 Ab alone had no significant effect on the percentage of sub-G1 events (Figure 1A, upper left panels), indicating that HCT116 cells are resistant to signals through CD95. Treatment with CHX alone had no reproducible effect on cell viability, but the combination of CHX with anti-CD95 Ab induced a significant increase in the percentage of sub-G1 events (Figure 1A, left panels). This increase in cell-death was almost completely abolished by prior incubation with an antagonist (non-signalling) anti-CD95 Ab.

A small increase in the percentage of sub-G1 events was observed 2 days after γ-irradiation with 16 Gy (Figure 1A, upper panels). As in unirradiated controls, the viability of irradiated cells was not affected by agonist anti-CD95 Ab alone, indicating that large-dose irradiation by itself does not sensitize these resistant cells to CD95 signals. A substantial increase in sub-G1 events was observed when radiation treatment was combined with CHX plus agonist anti-CD95 Ab, and this increase was larger than observed after treatment with CHX plus agonist anti-CD95 Ab in the absence of irradiation (Figure 1A).

Enhanced responsiveness was also observed when cells were examined for DNA strand breaks by TUNEL staining. Significant DNA breakage was observed in irradiated cells treated with CHX plus agonistic anti-CD95 Ab (Figure 1B), which was confirmed in a parallel DNA content analysis. TUNEL staining provided poorer resolution of microscopically confirmed CD95-induced carcinoma cell death than did DNA content analysis in these experiments. These results suggest that a large dose of γ-radiation can enhance the response of CD95-sensitized tumour cells to signals through CD95.

### Cumulative up-regulation of CD95 when γ-irradiation is fractionated into daily doses

One mechanism for enhancing the apoptotic response to CD95-mediated signalling may be through up-regulation of CD95 surface expression. CD95 expression is known to be regulated, in part, by the p53 protein (Owen-Schaub et al, 1995), and carcinoma cells exhibiting wild-type p53 activity were shown to up-regulate CD95 expression 8 h after a high dose of γ-rays (Sheard et al, 1997). However, clinical irradiation is usually fractionated into low doses, and it remained unknown whether surface expression of CD95 is sufficiently stable to accumulate during an extended

**Table 1** Surface CD95 expression in single-dose- or sequentially-irradiated cells expressing wild-type p53

| | MFI Index[a] | | | |
|---|---|---|---|---|
| **Cell lines** | **Untreated** | **16 Gy** | **Mock-irradiated (8×)** | **2 Gy (8×)** |
| HCT116 | 3.24 | 5.85 | 2.85[b] | 7.01[b] |
| MCF-7 | 1.80 | 5.42 | 1.63 | 5.19 |
| ZR-75.1 | 9.85 | 17.0 | 10.8 | 19.6 |

[a]Values for MFI Index were calculated as described in Materials and Methods. [b]Maintained in 1% FCS; other cells were maintained in 10% FCS.

© 1999 Cancer Research Campaign

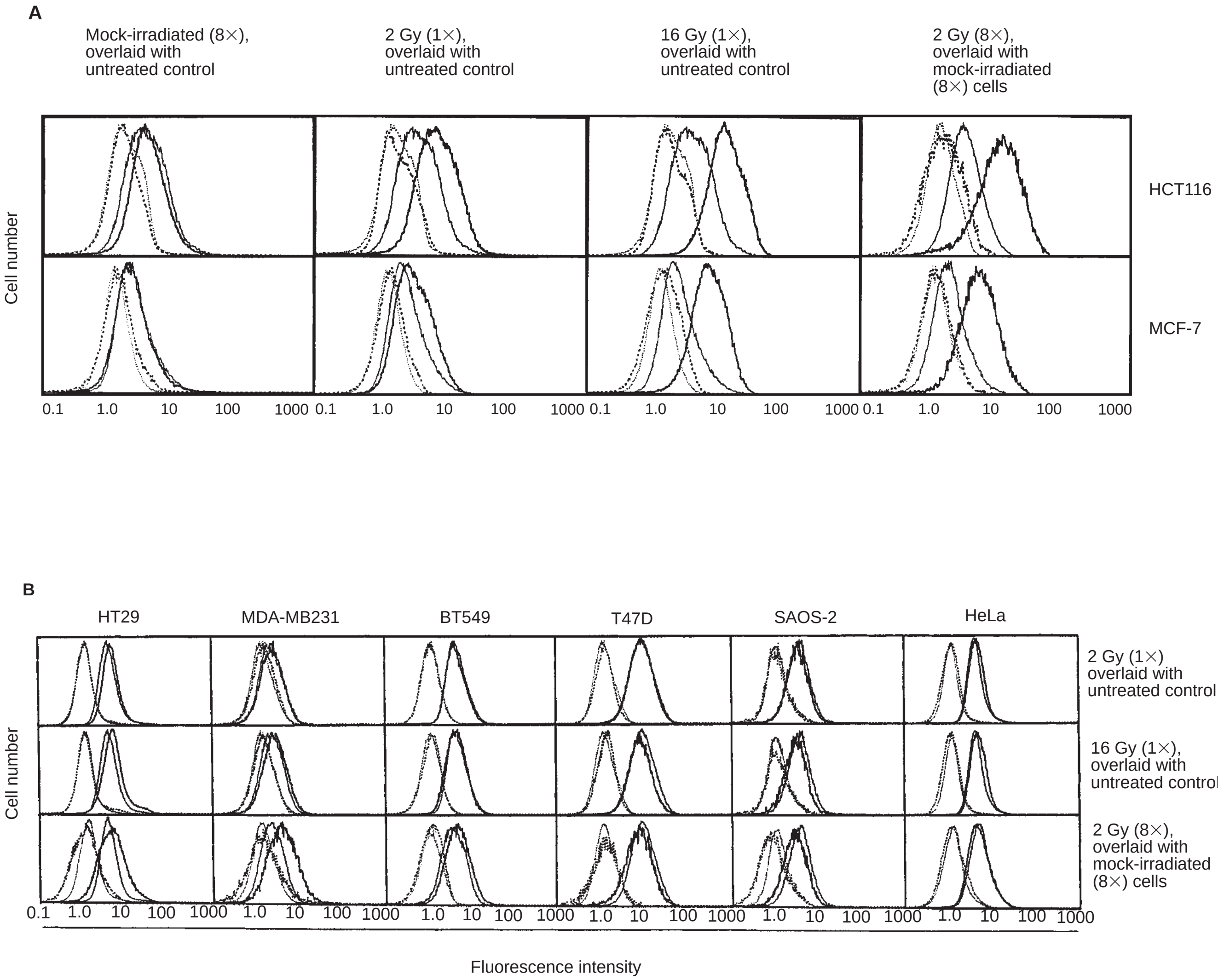

**Figure 2** Regulation of surface CD95 expression after γ-irradiation. Cells were treated (thick lines) with single-dose or fractionated irradiation. Controls (thin lines) were untreated or mock-irradiated. Cells were stained with anti-CD95 (solid lines) or irrelevant Ab (dotted lines) 1 day after completion of irradiation, and analysed by flow cytometry. (**A**) HCT116 and MCF-7 cells, reported to express functional wild-type p53 activity. (**B**) Cell lines reported to express aberrant p53 activity. A minimum of three independent experiments was performed for each cell line course of daily fractionated treatment. To address this question, multiple cell lines with wild-type statuses of p53 were γ-irradiated with 2 Gy on 8 consecutive weekdays and examined 1 day later for CD95 expression levels. HCT116 and MCF-7 cells constitutively express a low level of CD95 on their cell surface, and this level is unaffected by repetitive mock-irradiation (Figure 2A, top panels). As a control, these cells were treated with a single dose of 2 Gy, resulting in moderate up-regulation of CD95, or with 16 Gy, resulting in significantly higher CD95 expression (middle panels). Fractionation of the 16 Gy dose into 8 daily doses of 2 Gy (lower panels) resulted in an approximately equal enhancement of CD95 expression as obtained with the single large dose (Table 1). Similar results were obtained with ZR-75.1 cells (Table 1).

Six cell lines reported to exhibit aberrant p53-activity were also examined, to determine whether fractionated irradiation might affect CD95 expression in the large subset of cancer cells that possess abnormal p53 activity. A single dose of 16 Gy induced slight up-regulation in HT29 cells, but not in the other five cell lines examined (Figure 2B). Dose-fractionated irradiation reproducibly induced slight up-regulation in two lines (HT29 and MDA-MB231), but had no significant effect on CD95 expression in the remaining four lines. All cell lines were able to up-regulate CD95 after treatment with interferon-γ, regardless of p53 status (data not shown). Taken together, these results indicate that up-regulation of CD95 is cumulative during fractionated γ-irradiation, but abundant up-regulation is limited to those cells bearing normal wild-type p53.

*© 1999 Cancer Research Campaign*

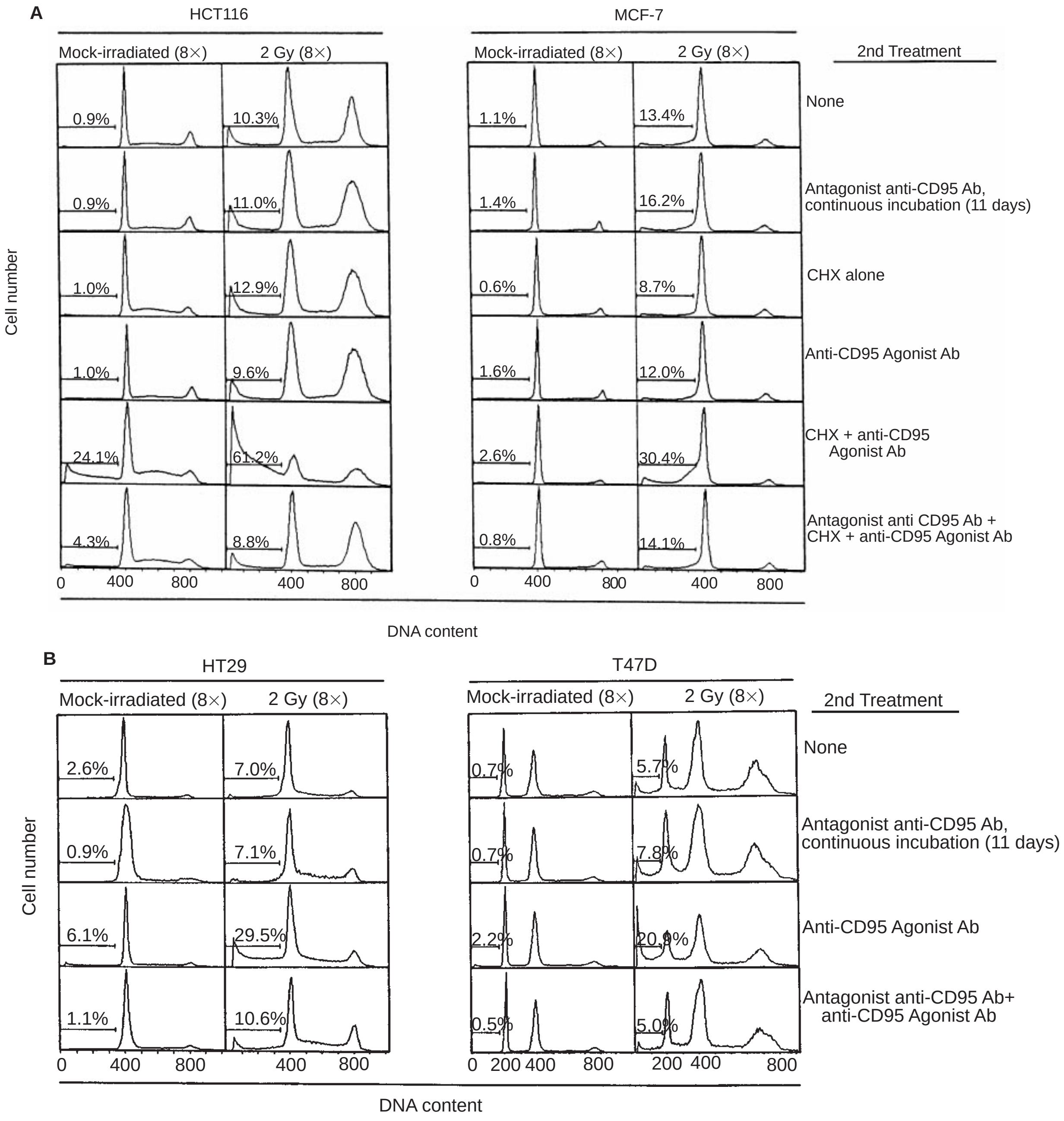

**Figure 3** Effect of CD95-mediated signalling on viability of carcinoma cells treated with fractionated γ-irradiation. Cells were irradiated with 2 Gy γ-rays daily on 8 consecutive weekdays. (**A**) CD95-resistant cell lines HCT116 and MCF-7. Eight hours after the final irradiation, cells were pretreated with CHX (10 μg $ml^{-1}$), treated with agonist anti-CD95 Ab (1 μg $ml^{-1}$), or both. Cells were harvested 20 h later, 42 h after the final exchange of medium, and analysed as in Figure 1A. (**B**) Partially-sensitive cell lines HT29 and T47D, treated with agonist anti-CD95 Ab (0.2 μg $ml^{-1}$) in the absence of CHX. Specific groups were subject to continuous incubation with antagonist anti-CD95 Ab throughout the course of fractionated irradiation, as shown. T47D cells contained substantial populations with approximately tetraploid and octaploid numbers of chromosomes. The lowest G1 peak of T47D cells was positioned at channel 200 to permit viewing of near-octaploid cells. Results are representative of three independent experiments for each cell line

### Response to signals through CD95 after fractionated γ-irradiation

To examine whether fractionated radiation treatment enhances the response of CD95-resistant carcinoma cells to CD95-mediated signals, HCT116 and MCF-7 cells were γ-irradiated with 2 Gy on 8 consecutive weekdays. Eight hours after completion of fractionated irradiation, cells were treated with CHX and/or agonist anti-CD95 Ab, incubated another 20 h, and examined for DNA content. Incubation with agonist anti-CD95 Ab alone did not alter the percentage of sub-G1 events in either mock-irradiated or sequentially-irradiated cells, indicating that fractionated irradiation did not overcome the inherent resistance of these tumour cells to CD95-mediated signals (Figure 3A). Incubation with agonist anti-CD95 Ab in the presence of CHX resulted in a substantially larger increase in the percentage of sub-G1 events in sequentially-irradiated cells than in identically treated mock-irradiated cells (Figure 3A). Prior incubation with antagonist anti-CD95 Ab specifically

© 1999 Cancer Research Campaign

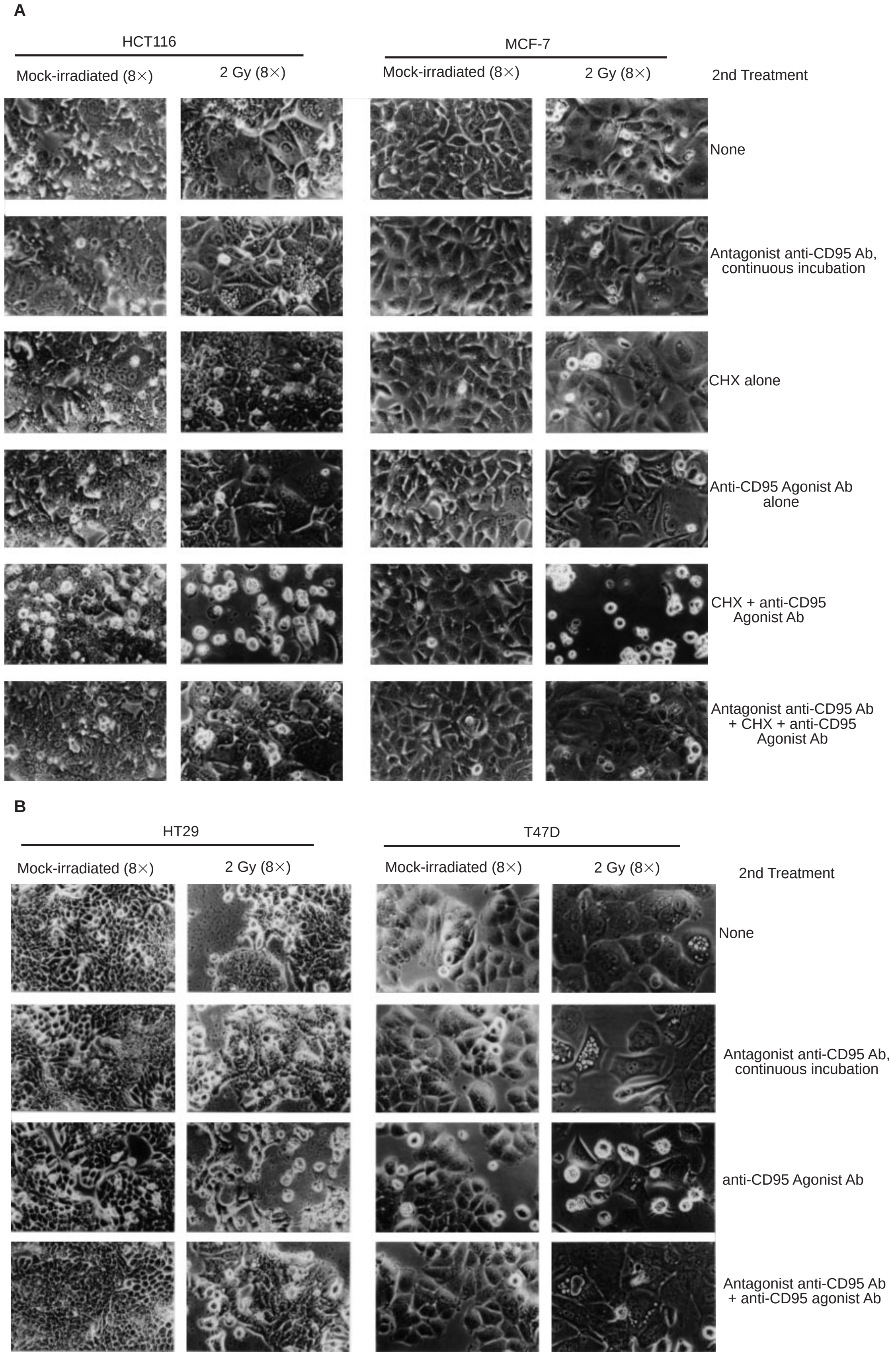

**Figure 4** Morphology of sequentially-irradiated or unirradiated cells continuously incubated with antagonist anti-CD95 Ab. Cell cultures were treated as in Figure 3. Adherent cells appear dark gray, detached cells appear round and bright. (**A**) CD95-resistant cell lines HCT116 and MCF-7. (**B**) Partially-sensitive cell lines HT29 and T47D. Original magnification ×320

*© 1999 Cancer Research Campaign*

inhibited induction of sub-G1 events. Thus, HCT116 and MCF-7 cells were rendered more responsive to CD95-mediated signals by fractionated irradiation, but only when supplied sensitizing pretreatment (e.g. CHX).

To extend these observations, two partially-sensitive carcinoma cell lines that do not require sensitizing pretreatment were examined. HT29 and T47D cells undergo a modest degree of apoptosis in response to treatment with agonist anti-CD95 Ab alone, but only after relatively long incubation times. Incubation with agonist Ab induced a small increase in sub-G1 events in mock-irradiated cells, but a significantly larger increase was reproducibly observed after identical treatment of sequentially-irradiated cells (Figure 3B). These data indicate that fractionated γ-irradiation alone is sufficient to enhance the vulnerability of partially-sensitive carcinoma cells to CD95-mediated apoptosis. Since HT29 and T47D cells express mutant p53 and exhibit little or no up-regulation of CD95 after fractionated irradiation (Figure 2B), the observed enhancement of responsiveness to CD95-mediated signals was independent of wild-type p53 status and CD95 up-regulation.

The T47D cells in our experiments contained subpopulations with approximately tetraploid (4N) and octaploid (8N) numbers of chromosomes (Figure 3B, channels 400 and 800), as previously reported (Graham et al, 1989). Following fractionated irradiation, an apparent accumulation of T47D and HCT116 cells in peaks containing 4N was observed (Figure 3 A, B). This accumulation was due, at least in part, to irradiation-induced generation of polyploidy, since these cells accumulated near-octaploid and eventually near-16N populations after fractionated irradiation (data not shown), and should not be interpreted as indicative of G2 cell-cycle arrest at these low doses.

To investigate whether autologous CD95/CD95-L signalling (Friesen et al, 1996; Fulda et al, 1997; Müller et al, 1997) might be involved in radiation-induced death in the model studied here, sequentially-irradiated cells were continuously incubated in the presence of antagonist anti-CD95 Ab throughout the 11 day protocol from the first dose of 2 Gy to harvest. Continuous culturing with antagonist anti-CD95 Ab did not prevent the formation of either radiation-induced sub-G1 events (Figure 3 A, B) or classical radiation-induced morphology involving cellular enlargement and vacuolation (Figure 4 A, B), even though the same concentration of antagonist Ab inhibited the formation of both sub-G1 events and morphological changes induced by agonist anti-CD95 Ab treatment. Relatedly, continuous inhibition of CD95/CD95-L interactions did not prevent radiation-induced polyploidy in T47D (Figure 3B) and HCT116 (data not shown) cells. Taken together, these results indicate that autologous CD95/CD95-L signalling did not mediate the damaging effects of fractionated γ-irradiation on the four cell lines examined.

### DISCUSSION

Many proteins are only transiently expressed in cells. For example, p53 expression increases 2–3 h after treatment of MCF-7 cells with ionizing radiation, but then rapidly declines (Lu and Lane, 1993). Transiently expressed proteins responding to cellular stress would not be expected to accumulate over time during daily dose-fractionation. However, evaluation of CD95 expression during fractionated γ-irradiation of $p53^{wild-type}$ cells reveals that fractionation of treatment into small daily doses results in strong up-regulation of CD95 expression levels, demonstrating that CD95 is sufficiently stable on the cell surface to allow accumulation during dose-fractionation. This supports the possibility that clinical radiotherapy of wild-type p53-expressing tumours may result in cumulative and prolonged elevation of CD95 expression, which may endure throughout the entire course (e.g. 30 fractions) of radiation therapy.

Daily fractionated γ-irradiation enhanced the response to CD95-mediated signals in four of four carcinoma cell lines. However, irradiation-induced increases in CD95 expression alone were not sufficient to sensitize CD95-resistant cells to CD95-mediated signals in the absence of CHX, and the responsiveness to CD95-mediated signals in two of the four cell lines was increased in spite of little or no detectable up-regulation of CD95, indicating that CD95 up-regulation is not a critical factor for enhancing the responsiveness to CD95-mediated signals. This latter finding is similar to a report that inhibition of protein export with brefeldin A prevents up-regulation of surface CD95 and yet sensitizes carcinoma cells to CD95-mediated signals (von Reyher et al, 1998). The question of whether, and to what extent, increases in CD95 expression can influence the apoptotic response to CD95-mediated signals has not been specifically addressed in this work. Notably, it has been reported that sensitization to CD95-mediated signalling can be obtained in CD95-resistant MCF-7 cells by genetically engineered overexpression of CD95 in the presence of protein A or sphingomyelinase (Jäättelä et al, 1995), leaving open the possibility that the magnitude of increased CD95 expression may influence the sensitivity of CD95-resistant cells.

The possibility that induction of tumour cell death by radiation treatment might involve autologous killing through CD95/CD95-L interactions has been suggested by the following observations: (i) ionizing radiation stimulates production of ceramide (Haimovitz-Friedman et al, 1994), (ii) γ-radiation-induced production of ceramide can induce up-regulation of CD95-L (Herr et al, 1997), (iii) disruption of the CD95/CD95-L signalling pathway in leukaemia cells inhibited ceramide-induced apoptosis (Herr et al, 1997). Furthermore, it was reported that autologous CD95/CD95-L signalling can be observed following cytotoxic treatments of some leukaemia, hepatoma and neuroblastoma cells (Friesen et al, 1996; Müller et al, 1997; and Fulda et al, 1997 respectively). However, inhibition of CD95/CD95-L interactions provided no protection from the lethal effect of daily fractionated γ-irradiation in the carcinoma cells studied here. Similarly, it has been previously reported that γ-irradiation (12 Gy) of a CD95-resistant subclone of Jurkat lymphoid cells induced cell death as efficiently as in CD95-sensitive parental cells (Eischen et al, 1997). The absence of autologous CD95/CD95-L signalling in the direct lethal effect of fractionated γ-irradiation on CD95-partially-sensitive cells suggests that the radiation-enhanced response to signals through CD95 might be fully exploitable using other modalites for ligating CD95. An additional sensitizing agent would be required to realize a similar benefit in CD95-resistant cells.

In summary, the present study indicates that CD95-partially-sensitive carcinoma cells, and also CD95-resistant cells which are pretreated with a sensitizing agent (e.g. CHX), become more responsive to CD95-mediated apoptotic signals after fractionated γ-irradiation. No evidence was found to suggest that autologous CD95/CD95-L signalling was active in the fractionated radiation-induced death of the four carcinoma cell lines tested. Since the CD95-independent nature of fractionated radiation-induced death was coupled with increased responsiveness to CD95-mediated signals, utilization of the heightened responsiveness of these irradiated carcinoma cells to CD95-mediated signalling probably requires an exogenous source of CD95 ligation.

© 1999 Cancer Research Campaign

### ACKNOWLEDGEMENTS

The authors wish to thank D Valik and J Borst for critically reading the manuscript, J Pecina and J Simicek for assistance with irradiation, and M Krasensky for his expertise. This work was supported by grants from the Czech IGA 4401–3 and GACR 301/96/K047.

### REFERENCES

Ashkenas J and Werb Z (1996) Proteolysis and the biochemistry of life-or-death decisions. *J Exp Med* **183**: 1947–1951

Clarke AR, Purdie CA, Harrison DJ, Morris RG, Bird CC, Hooper ML and Wyllie AH (1993) Thymocyte apoptosis induced by p53-dependent and independent pathways. *Nature* **362**: 849–852

Dhein J, Walczak H, Baeumler C, Debatin KM and Krammer PH (1995) Autocrine T-cell suicide mediated by APO-1 (Fas/CD95). *Nature* **373**: 438–441

Eischen CM, Schilling JD, Lynch DH, Krammer PH, and Leibson PJ (1996) Fc receptor-induced expression of Fas ligand on activated NK cells facilitates cell-mediated cytotoxicity and subsequent autocrine NK cell apoptosis. *J Immunol* **156**: 2693–2699

Eischen CM, Kottke TJ, Martins LM, Basi GS, Tung JS, Earnshaw WC, Leibson PJ, and Kaufmann SH (1997) Comparison of apoptosis in wild-type and Fas-resistant cells: chemotherapy-induced apoptosis is not dependent on Fas/Fas ligand interactions. *Blood* **90**: 935–943

Friesen C, Herr I, Krammer PH and Debatin KM (1996) Involvement of the CD95 (APO-1, Fas) receptor/ligand system in drug-induced apoptosis in leukemia cells. *Nat Med* **2**: 574–577

Fulda S, Sieverts H, Friesen C, Herr I, and Debatin KM (1997) The CD95 (APO-1/Fas) system mediates drug-induced apoptosis in neuroblastoma cells. *Cancer Res* **57**: 3823–3829

Graham ML, Dalquist KE and Horwitz KB (1989) Simultaneous measurement of progesterone receptors and DNA indices by flow cytometry: analysis of breast cancer cell mixtures and genetic instability of the T47D line. *Cancer Res* **49**: 3943–3949

Haimovitz-Friedman A, Kan CC, Ehleiter D, Persaud RS, McLoughlin M, Fuks Z and Kolesnick RN (1994) Ionizing radiation acts on cellular membranes to generate ceramide and initiate apoptosis. *J Exp Med* **180**: 525–535

Herr I, Wilhelm D, Böhler T, Angel P and Debatin KM (1997) Activation of CD95 (APO- 1/Fas) signaling by ceramide mediates cancer therapy-induced apoptosis. *EMBO J* **16**: 6200–6208

Irmler M, Thome M, Hahne M, Schneider P, Hofmann K, Steiner V, Bodmer JL, Schroter M, Burns K, Mattmann C, Rimoldi D, French LE and Tschopp J (1997) Inhibition of death receptor signals by cellular FLIP. *Nature* **388**: 190–195

Jäättelä M, Benedict M, Tewari M, Shayman JA and Dixit VM (1995) Bcl-X and Bcl-2 inhibit TNF and Fas-induced apoptosis and activation of phospholipase $A_2$ in breast carcinoma cells. *Oncogene* **10**: 2297–2305

Krammer PH, Dhein J, Walczak H, Berhmann I, Mariani S, Matiba B, Fath M, Daniel PT, Knipping E, Westendorp MO, Stricker K, Bäumler C, Hellbardt S, Germer M, Peter ME and Debatin KM (1994) The role of APO-1 mediated apoptosis in the immune system. *Immunol Rev* **142**: 175–191

Landowski TH, Gleason-Guzman MC and Dalton WS (1997a) Selection for drug resistance results in resistance to Fas-mediated apoptosis. *Blood* **89**: 1854–1861

Landowski TH, Qu N, Buyuksal I, Painter JS and Dalton WS (1997b) Mutations in the Fas antigen in patients with multiple myeloma. *Blood* **90**: 4266–4270

Lichter AS and Lawrence TS (1995) Recent advances in radiation oncology. *N Engl J Med* **332**: 371–379

Lowe SW, Schmitt EM, Smith SW, Osborne BA and Jacks T (1993) p53 is required for radiation-induced apoptosis in mouse thymocytes. *Nature* **362**: 847–849

Lu X, and Lane DP (1993) Differential induction of transcriptionally active p53 following UV or ionizing radiation: defects in chromosome instability syndromes? *Cell* **75**: 765–778

Martinez-Lorenzo MJ, Gamen S, Etxeberria J, Lasierra P, Larrad L, Pineiro A, Anel A, Naval J and Alava MA (1998) Resistance to apoptosis correlates with a highly proliferative phenotype and loss of Fas and CPP32 (caspase-3) expression in human leukemia cells. *Int J Cancer* **75**: 473–481

Mori S, Jewett A, Murakami-Mori K, Cavalcanti M and Bonavida B (1997) The participation of the Fas-mediated cytotoxic pathway by natural killer cells is tumor-cell-dependent. *Cancer Immunol Immunother* **44**: 282–290

Müller M, Strand S, Hug H, Heinemann EM, Walczak H, Hofmann WJ, Stremmel W, Krammer PH and Galle PR (1997) Drug-induced apoptosis in hepatoma cells is mediated by the CD95 (APO-1/Fas) receptor/ligand system and involves activation of p53. *J Clin Invest* **99**: 403–413

Nagata S (1997) Apoptosis by death factor. *Cell* **88**: 355–365

Oshimi Y, Oda S, Honda Y, Nagata S and Miyazaki S (1996) Involvement of Fas ligand and Fas-mediated pathway in the cytotoxicity of human natural killer cells. *J Immunol* **157**: 2909–2915

Owen-Schaub LB, Zhang W, Cusack JC, Angelo LS, Santee SM, Fujiwara T, Roth JA, Deisseroth AB, Zhang W-W, Kruzel E and Radinsky R (1995) Wild-type human p53 and a temperature sensitive mutant induce Fas/APO-1 expression. *Mol Cell Biol* **15**: 3032–3040

Sheard MA, Vojtesek B, Janakova L, Kovarik J and Zaloudik J (1997) Up-regulation of Fas (CD95) in human $p53^{wild\text{-}type}$ cancer cells treated with ionizing radiation. *Int J Cancer* **73**: 757–762

Suda T, Okazaki T, Naito Y, Yokota T, Arai N, Ozaki S, Nakao K and Nagata S (1995) Expression of the Fas ligand in cells of T cell lineage. *J Immunol* **154**: 3806–3813

von Reyher U, Sträter J, Kittstein W, Gschwendt M, Krammer PH and Möller P (1998) Colon carcinoma cells use different mechanisms to escape CD95-mediated apoptosis. *Cancer Res* **58**: 526–534

Williams BA, Makrigiannis AP, Blay J and Hoskin DW (1997) Treatment of the P815 murine mastocytoma with cisplatin or etoposide up-regulates cell-surface Fas (CD95) expression and increases sensitivity to anti-Fas antibody-mediated cytotoxicity and to lysis by anti-CD3-activated killer-T cells. *Int J Cancer* **73**: 416–423

Zhan Q, Fan S, Bae I, Guillouf C, Liebermann DA, O'Connor PM and Fornace AJ (1994) Induction of bax by genotoxic stress in human cells correlates with normal p53 status and apoptosis. *Oncogene* **9**: 3743–3751

© 1999 Cancer Research Campaign
